# Fixation method influences FLASH skin sparing in an *in vivo* leg model

**DOI:** 10.2340/1651-226X.2025.43972

**Published:** 2025-08-05

**Authors:** Line Kristensen, Cathrine Overgaard, Jacob Graversen Johansen, Anna Holtz Hansen, Niels Bassler, Per Rugaard Poulsen, Brita Singers Sørensen

**Affiliations:** a Danish Centre for Particle Therapy, Aarhus University Hospital, Aarhus, Denmark; b Department of Experimental Clinical Oncology, Aarhus University Hospital, Aarhus, Denmark; c Department of Clinical Medicine, Aarhus University, Aarhus, Denmark

**Keywords:** FLASH, ultra-high dose rate, normal tissue sparing, acute toxicity, murine model

## Abstract

**Background and purpose:**

The FLASH effect, where ultra-high dose rate elicits a favourable normal tissue-sparing, has been shown in several preclinical studies. Study setup differences, for example fixation methods that affect blood flow, can influence radiation response but are unexplored for FLASH. This study compared FLASH’s acute skin-sparing effect with two fixation methods: a glued fixation (no blood flow restriction) and taped fixation (slight blood flow restriction).

**Patient/material and methods:**

Female CDF1 mice were irradiated on their hind foot using a glue-fixation or tape-fixation method. Glue-fixated mice were only taped during the glueing procedure and had a 10-min unrestricted period afterwards before irradiation, while tape-fixated mice were taped shortly before and throughout irradiation. Mice received single-dose irradiation (19–58 Gy) with either conventional dose rate (CONV, protons 0.06 Gy/s, electrons 0.16 Gy/s) or FLASH (electrons, 223–233 Gy/s). Differences in skin toxicity were analysed.

**Results:**

CONV-treated tape-fixated mice required a 16–17% higher dose to induce skin toxicity relative to glued mice for both protons and electrons. Meanwhile, the fixation method did not affect FLASH-treated mice. The resulting electron FLASH-sparing effect was reduced by 18% due to the shift in radiosensitivity for CONV-treated mice.

**Interpretation:**

CONV-treated tape-fixated mice were more radioresistant than the glue-fixated mice, consistent with the expected response to mild hypoxia. FLASH-treated mice were unaffected. These findings demonstrate the impact of fixation and, in turn, oxygen level on the differential CONV versus FLASH skin response. The results highlight the importance of minimal systemic influence on animals during FLASH studies.

## Introduction

The FLASH effect, where ultra-high dose rate (UHDR) elicits a favourable normal tissue-sparing, has been shown in several preclinical studies, while the curative effect on cancer was maintained [1–8]. The sparing of FLASH has been investigated by comparing conventionally (CONV) low-dose-rate treated and UHDR-treated models, primarily using single fraction irradiations in several tissues. The murine skin model has been extensively used for FLASH studies [6, 9–12] and has recently been used to establish dose-response relationships after electron and proton FLASH irradiations [3, 10, 13, 14]. With the quick dose delivery, immobilisation of the animal model is crucial for target dose accuracy.

For unanaesthetised animals, the fixation of the target tissue needs to be secure enough that conscious animals cannot retract the tissue from the irradiation field. However, fixations should not induce changes in blood flow, as this can influence a tissue’s oxygen supply and, thereby, radiation response, making it more radioresistant [15]. Thus, fixations must balance the constraint of animals with minimal influence on blood flow. In murine irradiation studies by Sørensen et al. [3, 13] and Kristensen et al. [10, 14], the fixation of the target tissue was solved by a glue fixation of the target limb. The glue-fixation method is, however, primarily suitable for single-treatment use, as repeated glue-fixation procedures give a risk of skin damage.

With the recent movement towards fractionated experiments [16], an animal fixation method suitable for repeated use is needed. Tape-fixation of unanaesthetised mice has been used previously for fractionated experiments [17], and recently in a fractionated FLASH experiment (unpublished data). The tape fixation induces a risk of blood flow restriction, and the tissue might become locally hypoxic. As oxygen has been speculated to play a role in FLASH tissue sparing, as, for example seen with hyperoxia radio-sensitising skin to FLASH but not CONV irradiation [18], the hypoxia-induced radio-resistance may not be equal for CONV and FLASH-treated mice.

This study aimed to determine if fixation-induced changes in radiation response would have similar impact on a CONV-treated and FLASH-treated murine model. The study compared FLASH’s acute skin-sparing effect after single-fraction irradiation under two different fixation methods: a glued fixation (unimpeded blood flow) and a taped fixation (blood flow restriction). The study hypothesised that fixation would influence radio-sensitivity and that the tape-induced radio-resistance would not be equal for CONV and FLASH treatment.

## Material and methods

### Study overview

This study experimentally compared two fixation methods under conventional dose rate proton beam irradiation, followed by a retrospective analysis of preclinical animal studies that used either fixation method under electron irradiation [10] (unpublished data). The retrospective analysis in the electron beam study served to confirm findings from the proton beam study and to test the impact on FLASH treatment. All three studies used the same murine model and a consistent and reproducible experimental irradiation setup, allowing for comparative evaluation with a specific focus on the impact of fixation technique. The methodology is summarised next with an emphasis on fixation procedures. For full experimental details, see Kristensen et al. [10].

### Murine model

All experiments used female C3D2F1 (CDF1) mice (11–19 weeks old) obtained from Janvier Labs (Le Genest-Saint-Isle, France). The mice were randomly assigned to treatment groups within each fixation method, with each animal considered an experimental unit. All experiments were approved by the Danish Animal Experiments Inspectorate (permits: 2017-15-0201-01218 and 2022-15-0201-01110) and conducted in compliance with the ARRIVE guidelines [19].

### Fixation method

Unanaesthetised mice were immobilised in custom-designed jigs corresponding to their assigned fixation method. Each jig consisted of a ventilated hollow tube for the mouse body and PMMA plates to support the right hindlimb during fixation.

**Figure 1:**
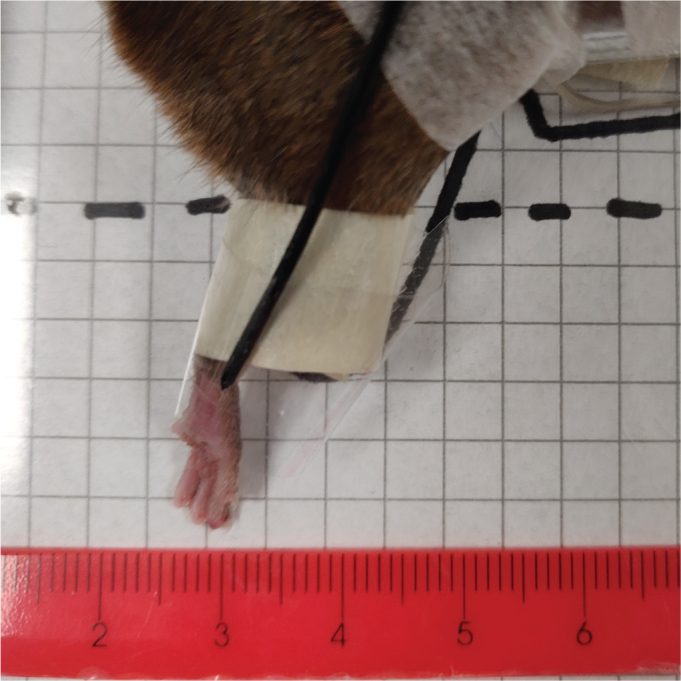
Target position example for a glued leg. The checkered template and ruler were used for water column depth determination.

For glued fixation, the hindlimb was supported by a single plate running along the inner thigh, ending near the ankle (Figure 1). A small droplet of histoacrylic glue was applied at the contact point between the plate and the inner thigh near the ankle joint to immobilise the leg. The ankle joint was aligned just beyond the plate’s lower edge. During the 5-min glue setting period, the leg was temporarily held in place with tape, followed by a minimum 10-min restitution period with the tape loosened. The leg was released immediately post-irradiation. Target positioning was standardised and determined using template pictures from an excerpt of previous studies (Figure 1).

**Figure 2:**
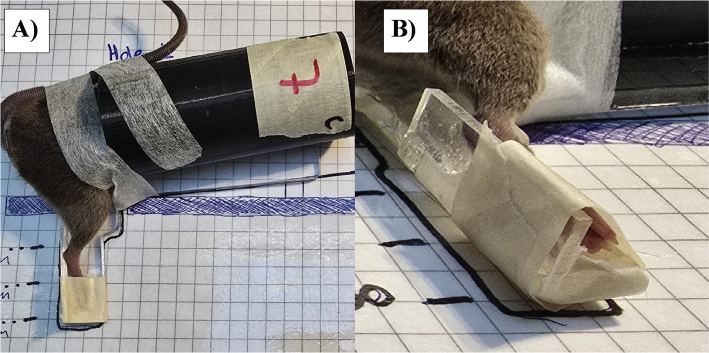
Depictions of a tape-fixated mouse. (A) Target position example for a taped leg. The restrained mouse is in a jig with its right foot fixated. The checkered template was used for water column depth determination. (B) Plantar view of the fixated mouse. The loose tape around the foot forms a triangle with air between the tape and the toes.

For taped fixation, two supporting polymethyl methacrylate (PMMA) plates were used: one along the inner thigh extending to the toes and one perpendicular to support the sole. The hindlimb was fixated immediately before irradiation with a strip of tape loosely tied around the toes and the supporting plate, creating a triangular space around the foot (Figure 2B). The toes were aligned with the lower edge of the supporting plate. As with the glued fixation, the leg was released immediately post-irradiation. Likewise, the target position was determined through template pictures (Figure 2A).

To assess potential differences in limb position between the two fixation types, a subset of fixation images was analysed to compare foot placement.

### Irradiations

For the proton beam study, two mouse cohorts were irradiated using either glued or taped fixation in the middle of a spread-out Bragg peak of a horizontal pencil beam scanning proton beamline (ProBeam, Varian, a Siemens Healthineers Company, Palo Alto, CA, USA) at the Danish Centre for Particle Therapy. Mice were treated using conventional low dose rates (CONV) of 0.06 Gy/s with five dose groups of 5–9 mice receiving single doses between 26 and 42 Gy. Treatment plans were made with the Eclipse treatment planning system (Varian Medical Systems, Palo Alto, CA, USA), and were verified dosimetrically with a plane-parallel and a thimble ionisation chamber. Dosimetric details are described in Overgaard et al. [20].

For the electron beam study, a retrospective analysis was conducted on preclinical studies with either a glued [10] or a taped (unpublished data) fixation. The mice were irradiated using a horizontal 16 MeV electron beam on a FLASH-enabled linac (TrueBeam accelerator, Varian, a Siemens Healthineers Company, Palo Alto, CA, USA). Mice received either CONV (0.16 Gy/s) or FLASH (taped: 223 Gy/s, glued: 233 Gy/s) dose rates, with groups of 6–10 mice receiving single doses between 19 and 58 Gy [10]. The absolute dose in the target position was measured with a diamond detector (flashDiamond, PTW Freiburg). CONV-doses were corrected for weekly output variations. FLASH doses were determined from Bergoz ACCT current transformer output and corrected for the dose at the target position. Dosimetric details of the electron beam setup are described in Kristensen et al. [10].

### Data collection and analysis

Acute skin damage was assessed daily from days 9 to 28 post-irradiation. Toxicity was graded as 2.5, 3.0, and 3.5 based on hair loss, moist desquamation area, and toe visibility. Observers were blinded to treatment and previous grades. Grading data were binarised using an automated R script to ensure analysis blinding [21]. Dose-response relationships were modelled using logistic regression, from which TD_50_ values (dose to elicit a toxic response in 50% of mice) were derived. Statistical differences in TD_50_ values between fixation groups were analysed and visualised using GraphPad Prism with multiple comparison t-tests across the three toxicity grades [22].

## Results

Target positioning in the water column was comparable between fixation groups (glue mean ± SD = 2.68 ± 0.15 cm (*n* = 82), tape mean ± SD = 2.81 ± 0.14 cm (*n* = 231)), reinforcing consistent setup conditions with the same doses delivered for both fixation types.

**Figure 3:**
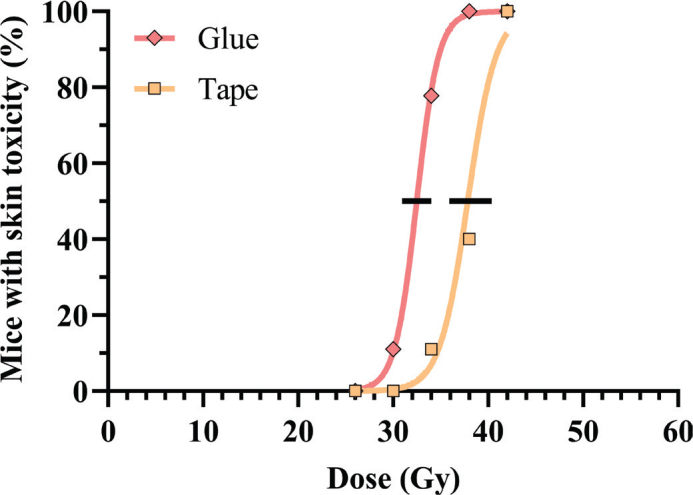
Dose–response relationship for acute skin toxicity (Grade 3) for mice treated with CONV protons during glued (red) or taped (orange) fixation. Black lines span the 95% confidence interval for 50% toxicity.

In the proton beam study of conventionally treated mice (*n* = 66), tape fixation shifted dose-response curves to higher doses, showing that higher doses were needed to induce skin damage with tape fixation. This shift was reflected in the TD_50_-values with 32.5 Gy for glued mice and 37.9 Gy for taped mice (Figure 3). The fixation method displayed a 17% difference under conventional proton irradiation, with reduced radiosensitivity for mice restrained with tape.

**Figure 4:**
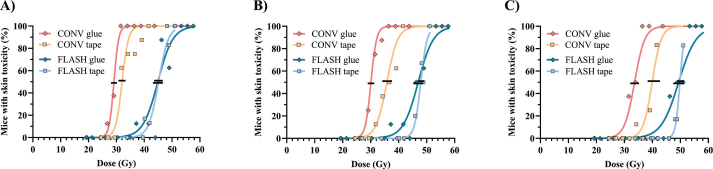
Dose–response relationship for mice treated with conventional (CONV, red nuances) and FLASH (blue nuances) electron irradiation. Black lines span the 95% confidence interval for 50% toxicity. (A) Moderate acute skin toxicity equalling grade 2.5, (B) Severe acute skin toxicity equal grade 3.0, (C) Severe acute skin toxicity equal grade 3.5. The separate curves for glued mice can be found in Kristensen et al. [10]

**Figure 5:**
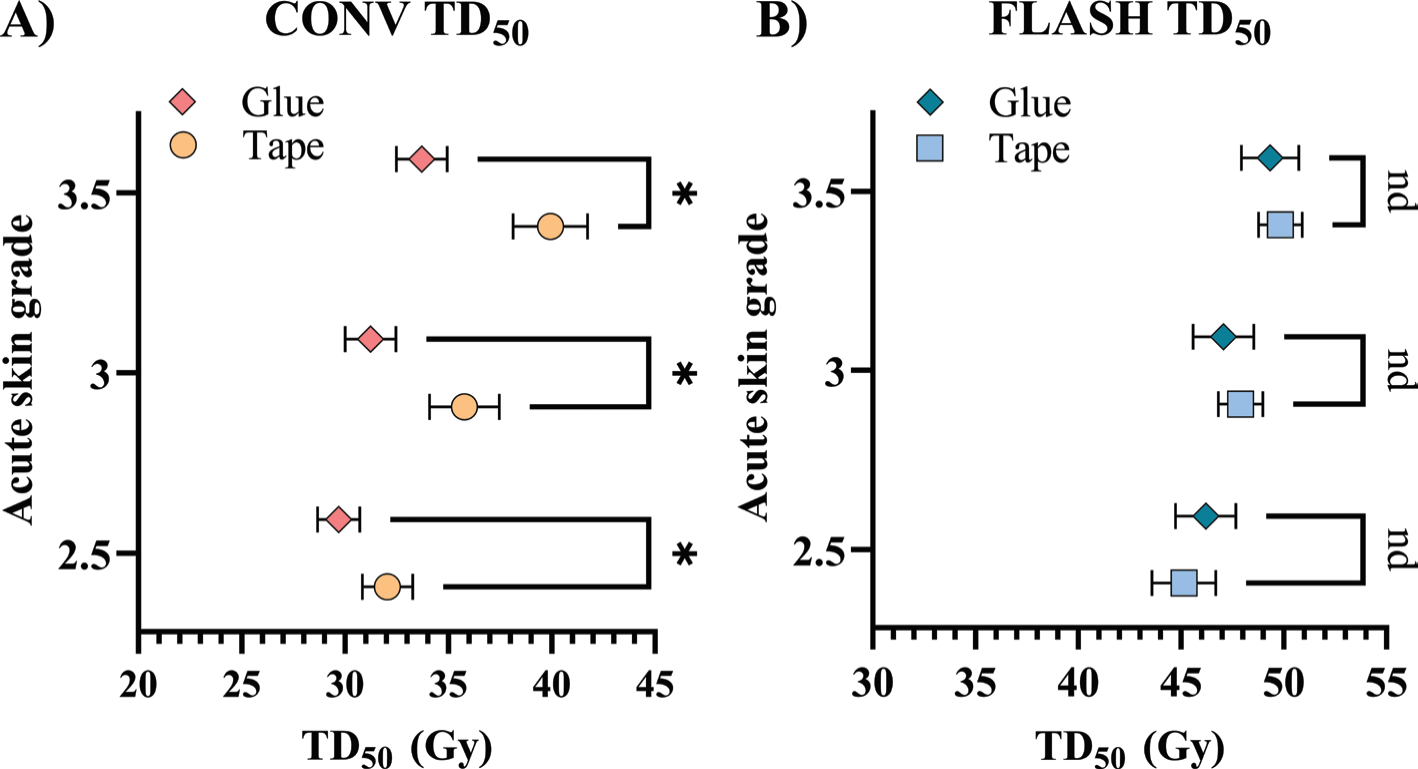
Dose to elicit a response in 50% of mice (TD_50_) in each acute skin grade for glued- and tape-fixated electron irradiated mice. Each dot represents the mean TD_50_, with error bars being standard error of the mean (SEM). * highlights a significant difference between means (*p* < 0.05) and highlights no significant difference. (A) For mice treated with a conventional dose rate. (B) For mice treated with FLASH.

These findings prompted a comparison as to whether a similar fixation-induced difference existed in previously reported data for electron-irradiated mice, comparing glued mice [10] and taped mice (unpublished data). Similar to the proton beam study, the electron-irradiated mice with CONV dose rate showed tape fixation to produce a rightward shift in the dose-response curve (Figure 4), with a higher dose needed to induce skin damage. A significantly higher TD_50_ was found for tape-fixated mice compared to glued mice for CONV across all three toxicity grades (Figure 5A). The average difference in CONV TD_50_ was 16%, consistent with the proton beam study findings. In contrast, for FLASH-irradiated mice, dose-response curves and TD_50_ values did not significantly differ by fixation method (Figures 4 and 5B).

The mean dose-modification factor (DMF) of electron beam FLASH was 1.51 (range 1.45–1.54) for glued mice [10] and 1.33 (1.25–1.41) for taped mice, indicating an 18% reduction in observed FLASH effect solely due to changes in the CONV baseline.

## Discussion and conclusion

This study examined how two leg fixation methods, glue and tape, affected radiation-induced skin toxicity in both conventional (CONV) and ultra-high dose rate (FLASH) settings. The acute skin toxicity in response to irradiation was changed with different fixation methods for CONV-treated but not FLASH-treated mice. Despite only changing the fixation method from glue to tape, the electron FLASH skin sparing, the DMF, was reduced by 18% from 1.51 (DMF range 1.45–1.54) with glue fixation [10] to 1.33 (DMF range 1.25–1.41) with tape fixation. The difference in DMF was due solely to changes in radio-sensitivity under CONV conditions (Figure 4); FLASH responses were unaffected by fixation method. FLASH TD_50_ values were 1% different between fixation methods, with completely overlapping 95% confidence intervals (Table 1) and SEM (Figure 5B). Meanwhile, CONV TD_50_ had a 16% (range 10–19%) difference with non-overlapping 95% confidence intervals (Table 1) and SEM (Figure 5A).

**Table 1:** An overview of TD_50_ and the percentage difference between the fixation methods within each dose rate. Electron beam study TD_50_ values were directly derived from Kristensen et al. [10].

PROTON BEAM STUDY – CONV			
**Toxicity score**	**CONV TD_50_ Glue (Gy)**	**CONV TD_50_ Tape (Gy)**	**CONV TD_50_ Ratio**
Grade 3.0	32.5 (30.9–34.0)	37.9 (35.9–40.4)	1.17
ELECTRON BEAM STUDY – CONV			
**Toxicity score**	**CONV TD_50_ Glue (Gy)** [10]	**CONV TD_50_ Tape (Gy)**	**CONV TD_50_ Ratio**
Grade 2.5	29.1 (28.1–30.2)	32.1 (30.8–33.3)	1.10
Grade 3.0	30.1 (29.0–31.3)	35.8 (34.1–37.6)	1.19
Grade 3.5	33.7 (32.1–35.2)	39.9 (38.3–42.7)	1.18
Mean			1.16
ELECTRON BEAM STUDY – FLASH			
**Toxicity score**	**FLASH TD_50_ Glue (Gy)** [10]	**FLASH TD_50_ Tape (Gy)**	**FLASH TD_50_ Ratio**
Grade 2.5	44.9 (43.2–46.7)	45.2 (43.6–46.7)	1.01
Grade 3.0	47.0 (45.2–48.8)	47.9 (46.7–49.1)	1.02
Grade 3.5	49.4 (47.5–51.4)	49.9 (48.9–51.4)	1.01
Mean			1.01

CONV-treated tape-fixated mice were more radioresistant than the glue-fixated mice. The CONV TD_50_ comparisons (Figures 3 and 5A) showed that taped mice needed significantly higher doses to achieve similar skin damage. The response after tape fixation relative to glue fixation was similar for the proton-irradiated (17%) and the electron-irradiated mice (16%). The similarity in fixation-induced radio-resistance across both modalities points towards a general tendency for similar blood flow restriction with the fixation method across the studies.

A major strength of this work is the consistency across the experimental conditions, including the same facility, water bath setup, mouse strain, mouse age, and the same observers, minimising confounding factors. Furthermore, the repeated testing of the fixation method across modalities with similar results highlights the fixation’s robustness and repeatability. The near-identical target placement for both fixations (mean 0.13 cm difference) further confirmed experimental consistency. However, the absence of direct physiological measurements, such as oxygen perfusion, limits the mechanistic interpretation. Future studies incorporating these measurements, or changing blood supply substantially, could clarify the role of blood supply and oxygen in fixation-induced radiosensitivity changes and FLASH sparing in general. The influence of substantial blood flow restriction on FLASH sparing is currently under investigation in a similar model, including tissue oxygen measures.

The similar radio-resistance observed in CONV-treated tape-fixed mice across both proton and electron modalities suggests a general mechanism, possibly involving reduced blood flow or local hypoxia due to the mechanical pressure of the tape. The effect must be due to a factor whose presence is influential *during* the irradiation. This hypothesis is seen in the light of Schwartz’s classic study [12] demonstrating reduced skin response when a radioactive source was tightly bound to the skin: an effect later linked to hypoxia and the oxygen enhancement ratio [23]. Although we did not directly measure blood flow or oxygen tension, the lack of a fixation effect under FLASH conditions supports the idea that oxygen somehow plays a role in FLASH sparing. If oxygen availability is already depleted or irrelevant during UHDR irradiation, mechanical changes in perfusion may not further influence the outcome. One hypothesis proposed on the mechanism of the FLASH sparing effect has been the influence of oxygen, for example, as an oxygen depletion [24–26] or oxygen-producing Reactive Oxygen Species (ROS) [27]. However, the hypotheses are highly debated [28].

As the mechanism behind FLASH tissue sparing is slowly unravelling, finding these steps for accurate study comparisons is crucial. Previous data have highlighted the influence of dose-sensitive endpoints on FLASH tissue sparing [12]. Another essential factor reported was the oxygen supply’s effect [29], where a complete blood supply restriction resulted in similar radiation response of CONV and FLASH treated mice, however within a limited dose range. This study adds observations that even small restrictions of blood flow and/or oxygen influence the CONV radiation response, but that an apparent change in FLASH dose-modification is not due to any change in response to FLASH. Furthermore, the study adds the fixation method to the list of key components to consider in across-study comparisons or highlights the difficulty in doing so.

In conclusion, the fixation method can substantially influence the perceived FLASH sparing effect, not by altering the response to UHDR itself, but by modifying the conventional baseline. Tape fixation increased the TD_50_ for CONV-treated mice by 16–17%, leading to an apparent 18% reduction in the FLASH dose modification factor. The fixation-induced shift in radio-sensitivity was consistent for CONV across protons and electrons, suggesting a shared underlying mechanism. These findings underscore the importance of standardised fixation protocols in preclinical radiation studies. Interestingly, the taped fixation did not induce any changed response in the FLASH-treated mice, suggesting that oxygen plays a role in the mechanism of FLASH, as even though there may be some degree of hypoxia induced, it does not alter the radiation response to FLASH.

## Data Availability

Data are available for sharing upon request to the corresponding author.
